# A Model for Allosteric Communication in Drug Transport by the AcrAB-TolC Tripartite Efflux Pump

**DOI:** 10.3390/antibiotics11010052

**Published:** 2022-01-01

**Authors:** Anya Webber, Malitha Ratnaweera, Andrzej Harris, Ben F. Luisi, Véronique Yvette Ntsogo Enguéné

**Affiliations:** 1 Department of Biochemistry, University of Cambridge, Tennis Court Road, Cambridge CB2 1GA, UK; anya.webber@costellomedical.com (A.W.); ams243@cam.ac.uk (A.H.); 2 Department of Oncology, MRC Weatherall Institute of Molecular Medicine, University of Oxford, Oxford OX3 9DS, UK; malitha.ratnaweera@oncology.ox.ac.uk

**Keywords:** allostery, antimicrobial resistance, conformational changes, efflux pump, energetic transition, gram-negative bacteria, pump activation

## Abstract

RND family efflux pumps are complex macromolecular machines involved in multidrug resistance by extruding antibiotics from the cell. While structural studies and molecular dynamics simulations have provided insights into the architecture and conformational states of the pumps, the path followed by conformational changes from the inner membrane protein (IMP) to the periplasmic membrane fusion protein (MFP) and to the outer membrane protein (OMP) in tripartite efflux assemblies is not fully understood. Here, we investigated AcrAB-TolC efflux pump’s allostery by comparing resting and transport states using difference distance matrices supplemented with evolutionary couplings data and buried surface area measurements. Our analysis indicated that substrate binding by the IMP triggers quaternary level conformational changes in the MFP, which induce OMP to switch from the closed state to the open state, accompanied by a considerable increase in the interface area between the MFP subunits and between the OMPs and MFPs. This suggests that the pump’s transport-ready state is at a more favourable energy level than the resting state, but raises the puzzle of how the pump does not become stably trapped in a transport-intermediate state. We propose a model for pump allostery that includes a downhill energetic transition process from a proposed ‘activated’ transport state back to the resting pump.

## 1. Introduction

Antimicrobial resistance rates are rising: it is predicted that by 2050 there could be ten million deaths per year due to drug-resistant infections [1]. Compounding the problem of high resistance rates, the development of new antimicrobials has stalled due to research and funding challenges [2,3]. Understanding the underlying mechanisms through which pathogens develop resistance is key to meeting this growing health challenge [4]. In gram-negative bacteria, an important multidrug resistance mechanism is the overexpression of drug efflux pumps [5]. These pumps can export diverse antibiotics, preventing them from reaching their cellular targets [6]. They also contribute to pathogenicity through extrusion of molecules involved in bacterial toxicity, quorum sensing, and biofilm formation [7]. Despite the wide range of existing pumps among bacteria showing diverse structure and activities, those systems share broad similarities including a potential requirement for allosteric switching. Their functional importance make them attractive targets for inhibitors design that either occlude or lock substrate binding sites or impede allosteric transitions [8].

Energy-dependent antibiotic efflux in bacteria was first identified more than forty years ago [9], and, to date, six families of bacterial transporters have been found to be involved in efflux [10]. These transporters are all localised in the inner membrane and—with the exception of the ATP-binding cassette (ABC) transporter family (which hydrolyses ATP)—all act as secondary transporters, using electrochemical gradients to drive substrate transport. Of the six identified families of bacterial transporters, members of the Resistance Nodulation Division (RND), Major Facilitator Superfamily (MFS), and ABC transporter families have all been found to form tripartite pump assemblies. A tripartite efflux pump, beside the inner membrane component, contains an adaptor membrane fusion protein (MFP), present in the periplasm, and an outer membrane protein (OMP). Together they make up a transport channel spanning from the cytoplasm to the extracellular medium. X-ray crystallography has helped to resolve individual components of efflux pumps, and the structures of whole pump assemblies in vitro have been determined by cryogenic electron microscopy (cryo-EM) [11,12,13,14]. Cryogenic electron tomography (cryo-ET) of whole bacterial cells has also provided images of these macromolecular machines in situ [15,16].

Inhibition of the tripartite efflux pumps presents a potential approach to increase the efficacy of existing antibiotics, but, so far, no clinically effective inhibitors have been developed [17]. Increased understanding of the structure, assembly, and mechanism of the pumps will help efforts to produce improved inhibitors, providing a valuable tool to tackling antimicrobial resistance.

**Figure 1:**
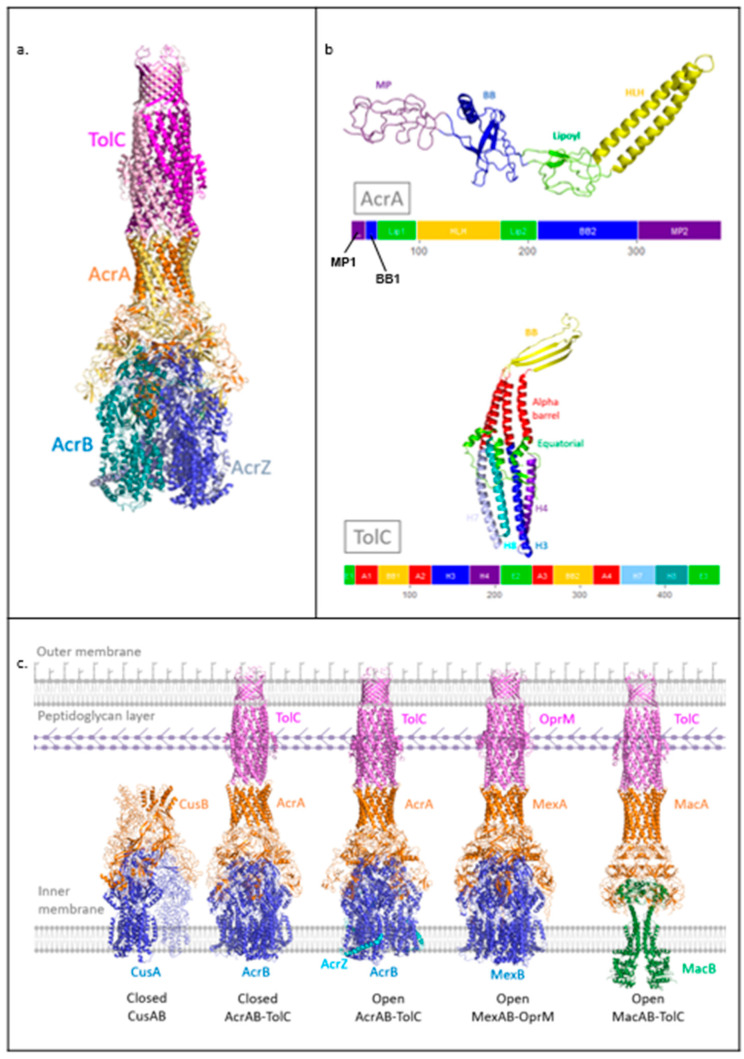
Structures of AcrAB-TolC and other drug efflux pump assemblies. (**a**) Transport state structure of AcrAB-TolC tripartite assembly (PDB: 5NG5). (**b**) Domain structure of AcrA and TolC protomers. For each protein, the top image shows the tertiary structure. The bottom image shows the primary sequence contribution to tertiary domain structure with equivalent colour scheme (5V5S). (**c**) High-resolution structures of RND family pump assemblies and of MacAB-TolC from the ABC transporter family (right to left: 3NE5, 5V5S, 5NG5, 6TA6, 5NIK). The CusAB trimer structure was generated from the PDB file of the monomeric unit through crystallographic symmetry using Coot [24,25]. The cell envelope includes a peptidoglycan layer and lipopolysaccharide in the outer leaflet of the outer membrane, both of which are represented schematically.

The AcrAB-TolC pump from *Escherichia coli* (Figure 1a) is part of the RND family and is one of the most well-characterised efflux pumps [18]. The assembly is formed by Acriflavine resistance protein B (AcrB) and A (AcrA) as well as tolerance to Colicins protein (TolC); these are the RND transporter, MFP, and OMP components, respectively. AcrB assembles as a trimer in the pump and substrates bind to the periplasmic poly-specific pocket in each of the three subunits [19]. Drug transport occurs via sequential conformational changes driven by a proton motive force (PMF) [20,21]. AcrB also associates with the small-factor AcrZ, which is thought to enhance extrusion of certain antibiotic classes through modulating the conformation of AcrB [22,23]. AcrA assembles as a hexameric ring in the tripartite pump to bridge AcrB with TolC [11]. AcrA protomers are found in two distinct conformations arranged in an alternating fashion around the ring. The AcrA protomer has four structural domains, each of which is generated from pairs of distinct segments of the amino acid chain (Figure 1b). The membrane proximal (MP) and beta barrel (BB) domains contact AcrB, with adjacent AcrA protomers forming distinct interactions with a single AcrB protomer, whilst the helix-loop-helix (HLH) domains form tip-to-tip interactions with the periplasmic helices of TolC. In the quaternary structure of the pump, the BB and lipoyl (Lip) domains assemble into two rings and HLH domains form a channel [11].

TolC assembles as a trimer, enclosing a channel that spans from the HLH domain of AcrA to the outer membrane. On its periplasm-facing surface, TolC contains the so-called equatorial domain, which interacts with the peptidoglycan layer in the cell wall [15]. The TolC protomer contains a structural repeat (Figure 1b), allowing the three sets of four periplasmic helices (H3, H4, H7, H8) to form quasi-equivalent interactions with the six HLH domains of AcrA [11]. The TolC helices situated between the outer membrane and the equatorial domain assemble into a unique alpha-barrel structure in the trimer which, along with the BB domain, forms a pore which is inserted into the outer membrane (Figure 1c).

Structures of assemblies of other RND family members have also been obtained in recent years, including the metal-ion transporter CusAB [26] and the drug efflux pump MexAB-OprM from *Pseudomonas aeruginosa* [27,28], (Figure 1c). Overall, these assemblies show similar organisation to AcrAB-TolC. The OMP TolC also interacts with other partner systems from the MFS and ABC superfamilies, such as ABC transporter MacB that energises the MacAB-TolC system, where the periplasmic protein MacA serves as the MFP (Figure 1c). MacA is engaged in tip-to-tip interactions with TolC, similarly to AcrA [29]. Evidence for the importance of MFPs, in particular the HLH domain for the formation of specific and functional pump assemblies via tip-to-tip interactions with the OMP, has also been provided by experiments showing that exchanging HLH domains between different chimeric MFPs to complement their cognate OMP can restore their functionality [30].

Currently, AcrAB-TolC is the only drug efflux pump for which there are high-resolution structures of the whole pump assembly in a resting and transport state, and these offer structural insight into the pump’s mechanism of action [13]. Molecular dynamics (MD) simulations have also been used to study AcrAB-TolC’s conformational change, particularly the functionally rotating mechanism of AcrB [31,32]. This was complemented by a recent study of numerous X-ray structures of AcrB with different substrates that revealed several intermediate states of the transport cycle [33]. Computational modelling of dynamics of the full tripartite assembly is difficult, partly due to the large size of the complex, which limits MD simulations to short timescales [34] that cannot capture larger-scale changes. Consequently, no simulations of the whole pump assembly have been reported to date [35]. On the other hand, biochemical studies have provided some insights into pump conformational changes. For example, mutational analysis identified key residues located in the helical regions of AcrA and TolC forming tip-to-tip interactions, which are critical for pore dilation on pump activation [36].

The current model for substrate extrusion by AcrAB-TolC describes the changes to AcrB on substrate binding, but lacks detail on how these changes are communicated to AcrA and TolC, as well as the role of the proton motive force (PMF) [18]. The model proposes a functionally rotating mechanism, where an AcrB subunit shifts from a Loose (L) to Tight (T) state on substrate binding, then transitions to an Open (O) state to release the drug into the MFP-OMP pore, and finally returns to the L state. This cycle is coupled to an alternating-access mechanism for proton transport that utilises PMF to drive drug efflux [35,37]. The proton relay network, made of several ionisable residues (D407, D408, K940, and R971) is located in the transmembrane domain of AcrB. The protonation/deprotonation of these residues triggers a collective motion of transmembrane helices through the LTO conformational cycle [37]. On substrate binding to AcrB, AcrA and TolC shift from a closed (resting) to an open (transport) conformation to allow drug extrusion, and then maintain the same open state throughout AcrB conformational cycling [13].

**Figure 2:**
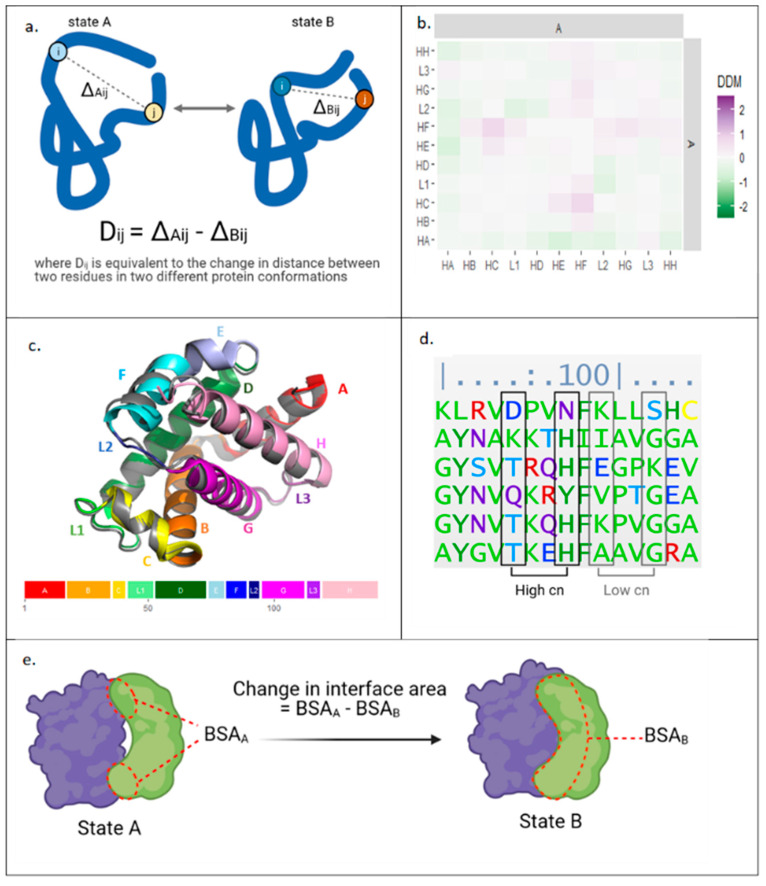
Bioinformatic approaches applied herein to study protein allostery. (**a**) Calculation of difference distance matrices (DDM) data from structures of a protein in two different conformations. (**b**) Block DDM for deoxy- and oxyhemoglobin-α. Squares represent average DDM changes for residues within domains specified. (**c**) Deoxyhemoglobin-α (grey, 1A3N) and oxyhemoglobin-α (multicoloured, 2DN1) aligned with C helices. Oxyhemoglobin-α’s colours correlate with domain structure shown below. (**d**) Calculation of evolutionary couplings: a section of a multiple sequence alignment for haemoglobin-α. (**e**) Calculation of the change in interface area at dimer interface. HA = helix A, L1 = loop 1, BSA = buried surface area, cn = coupling number, with higher values indicating stronger coupling.

Evaluating the communication of conformational changes in a complex multi-component system such as a tripartite assembly requires comparison of the different conformational states that is independent of reference-frame, and one approach suitable to this requirement is application of the difference distance matrix (DDM) [38]. DDM algorithms compute changes in distances between all residue pairs in two different protein states (Figure 2a). As an example, a block DDM analysis comparing the average movement of helices between deoxy- and oxy-states of the α-subunit of hemoglobin A is shown in Figure 2b. The plot clearly shows the movement of the F-helix, particularly in relation to the C-helix, which is known to be important for the protein’s mechanism of action [39]; whilst, the same motion is less discernible in the aligned structure models (Figure 2c).

In addition to structural data, sequence information can also provide clues into the allosteric mechanism. One approach is to compare sequences from evolutionarily related species, identifying evolutionary couplings (ECs) through sequence covariance in multiple sequence alignment (Figure 2d) [40]. The analysis assumes that residue pairs that are physically close or allosterically coupled are likely to be conserved to maintain functional interactions. Thus, potential allosteric residues can be identified using ECs data.

Furthermore, analysis of the allosteric mechanism can also be supported by estimating energies involved in conformational transitions, and one metric proportional to energy is change in buried surface area (Figure 2e). An increase in interface size between subunits indicates more interactions between residues, and so can be used as a proxy to suggest a more favourable energy state [41]. Comparing interface areas between conformers can provide information on the relative energy needed to transition between the observed states.

Here, we analysed available structural data to provide molecular insights into the long-distance subunit communication upon conformational changes in the efflux pump AcrAB-TolC. An analysis of the ECs in AcrA and TolC was performed alongside a DDM analysis to characterise the conformational changes of the pump and their evolutionary importance. To assess the energy required to transition between the resting and transport states of the pump, changes in interface area at subunit interfaces were measured. The results from these bioinformatic analyses were combined to propose an allosteric model for drug efflux in AcrAB-TolC.

## 2. Results

### 2.1. TolC Subunit Interface Contains Strongly Coupled Residue Pairs

To investigate allostery through an evolutionary lens, ECs data for TolC and AcrA were generated using multiple sequence alignments with ~60,000 and ~80,000 effective sequences for TolC and AcrA, respectively. EC analysis generates coupling numbers (cn) for each pair of residues, with higher cn values indicating stronger coupling. To reduce the risk of false positives, only residue pairs with coupling numbers (cn) above 1 were considered in further analysis. The remaining strongly coupled pairs were mapped onto molecular structure models, and residue pairs on the same helix were removed from the analysis, as these are unlikely to reflect allosteric interactions. Overall, we hypothesise that strongly coupled residues that are physically distant in the protein’s tertiary structure could be involved in allosteric communication [42,43].

**Figure 3:**
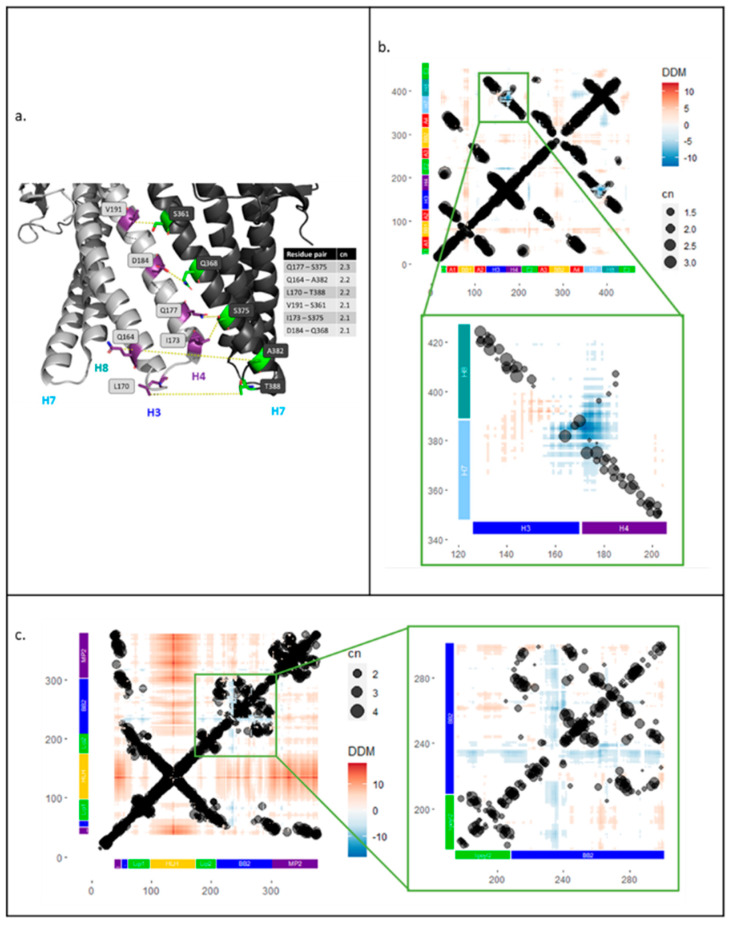
Strongly coupled residues at the interface between TolC subunits. Residue pairs in TolC identified from screen of evolutionary couplings data were filtered for couplings with cn ≥ 2 between residues in H3 and H4 (126–206) and residues in H7 and H8 (348–427) helices. (**a**) The cartoon shows helices of two TolC subunits in the transport-active open state with residue pairs between H3 and H4 helices of one protomer (light grey) and H7 helix of the second protomer (dark grey) (5NG5). Overlay of evolutionary couplings onto difference distance matrix analysis for TolC and AcrA. (**b**) Main: Difference distance matrix (DDM) and evolutionary couplings (EC) overlay for TolC protomer with domain structure on both axes. Inset: DDM and EC overlay for TolC periplasmic helices H3, H4, H7, H8. (**c**) Main: DDM and EC overlay for one AcrA subunit with domain structure on both axes. Inset: DDM and EC overlay for lipoyl and BB domains of AcrA.

As structural data show little movement of TolC above the equatorial domain [13], EC analysis focused on the periplasmic helices. Most coupled pairs within a TolC protomer were physically close to each other (less than 10 Å apart, data not shown) and so are likely involved in maintaining the tertiary fold of the protein rather than in transferring allosteric communication. However, some strongly coupled (cn ≥ 2) residues were found at the interface between TolC subunits (Figure 3a). These pairs likely play a role in the quaternary assembly of the TolC trimer: D184-Q368 and Q177-S375 could form polar interactions, and V191-S361, I173-S375 and L170-T388 could be part of hydrophobic interactions. Interestingly, we found two strongly coupled (cn > 2) residues, Q164 and A382, which were rather distant; they were separated by around 20 Å, both within a protomer and between neighbouring subunits, both in the resting and the transport state.

A similar filtering rationale was applied to the ECs data for AcrA. Due to the hairpin arrangement of the AcrA domains (Figure 1b), only residue pairs that were physically close within an AcrA protomer were identified (data not shown). For both the AcrA and TolC, the ECs data were combined with DDM analysis, yielding a more systematic mapping of the ECs onto the protein structures.

### 2.2. ECs Correlate to Intrasubunit Movements in TolC and AcrA

To investigate the conformational changes of AcrAB-TolC in relation to sequence covariance in the evolutionary couplings, DDM analysis of single TolC and AcrA protomers was performed and compared with the ECs data. For this analysis, structures of the proteins in resting and transport states were used. The average resolution of the structures was ~6 Å; therefore, any DDM movements of less than 3 Å were ignored in subsequent analysis. As in the previous step, ECs data were filtered to only use residue pairs with coupling numbers greater or equal to 1. The combined results were plotted, showing the relative movements and evolutionary couplings between different parts of the protein (Figure 3).

The DDM for TolC (Figure 3b) showed that, as previously reported [13], there was little TolC intrasubunit movement, with changes in the distance between residue pairs often being less than 2 Å. As expected, many of the strong ECs correlated to physically close residues, visible as lines of coupled residue pairs along the diagonal and as regions of strong ECs that correlate with the alpha barrel and equatorial domains of TolC (Figure 1b and Figure 3b). These did not show large movements in the DDM analysis. However, there was one region where the ECs and DDM appeared correlated: between residues 126–206 and 348–427, where the periplasmic helices of TolC moved closer to each other within a protomer. On closer inspection, these helix movements were slightly more complex, with simultaneous contraction and expansion (inset of Figure 3b). The largest region of contraction was between the H3-H4 loop and the H7-H8 loop; strong ECs were observed in this area, suggesting this movement might be conserved (Figure 3b, inset). Outward movement of helix H3 away from the loop of H7-H8 was also observed, but it did not correlate with any strong ECs (Figure 3b, inset).

The same approach was used to relate the DDM and ECs data for AcrA. A similar pattern of ECs that act to maintain the tertiary fold was observed with a line of coupled residues along the diagonal and strong couplings between the two sequence segments that fold back to make the halves of the AcrA domains (starting with MP1, BB1, Lipoyl 1, the turn at the hairpin of HLH domain, followed by Lipoyl 2, BB2, and MP2) (Figure 1b and Figure 3c). In our DDM analysis, AcrA appeared to be more dynamic than TolC with more extensive regions of movement (Figure 3c). The HLH domain, particularly the loop, had the largest outward movement, which is likely involved in widening of the AcrA pore for substrate extrusion, and enables it to form stronger tip-to-tip interactions with TolC. However, this movement does not correlate with any ECs. Some regions of contraction were observed, mainly in residues 230–240 in the BB domain, relative to the rest of the lipoyl and BB domains (Figure 1b). This region of contracted movement roughly colocalises with a region of strong ECs. However, upon closer inspection, the residues that move most were not strongly coupled (inset of Figure 3c).

### 2.3. TolC Periplasmic Helices Show Largest Outward Movement at the Quaternary Level

**Figure 4:**
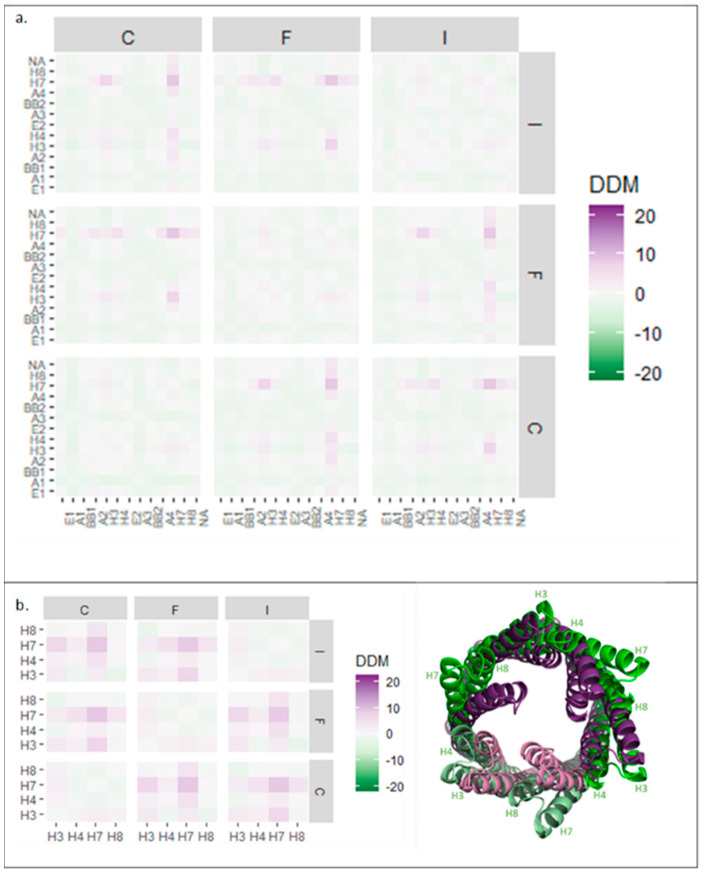
Block difference distance matrix (DDM) analysis of TolC domains in trimeric assembly. (**a**) Average DDM changes for all TolC domains in all three TolC protomers. (**b**) Left: Average DDM changes for TolC periplasmic helices H3, H4, H7, and H8 for all three TolC protomers. Right: TolC helices aligned in resting (purple, 5V5S) and transport (green, 5NG5) states, as viewed from above. Subunit numbering from PDB: 5NG5.

In the pump assembly, TolC protomers assemble into a trimer and do not just act individually. Therefore, the DDM analysis was expanded to probe quaternary changes by investigating the relative movements of protomers. To simplify the analysis, the average changes in each domain were calculated as a block (Figure 4).

The block analysis for all the TolC domains of all three protomers (Figure 4a) showed symmetrical conformational changes on pump activation. Quaternary changes were more pronounced than those at the tertiary level (stronger colouring on the squares off the diagonal) and an overall slight contraction of the trimer was observed (pale green colouring in the distribution) (Figure 4a). However, this contraction was relatively minor, illustrating that TolC was generally static, apart from the periplasmic helices, which moved outwards at the quaternary level (Figure 4b). Helix H7 moved the most—by more than 10 Å away from its neighbouring protomer’s equivalent helix (Figure 4b, right). As shown in Figure 3a, H7 formed an interface with H4 of the neighbouring subunit, so the movement of H7 is likely important for trimer assembly.

### 2.4. AcrA Domain Movements Corresponded to a Symmetrical Ring Contraction

**Figure 5:**
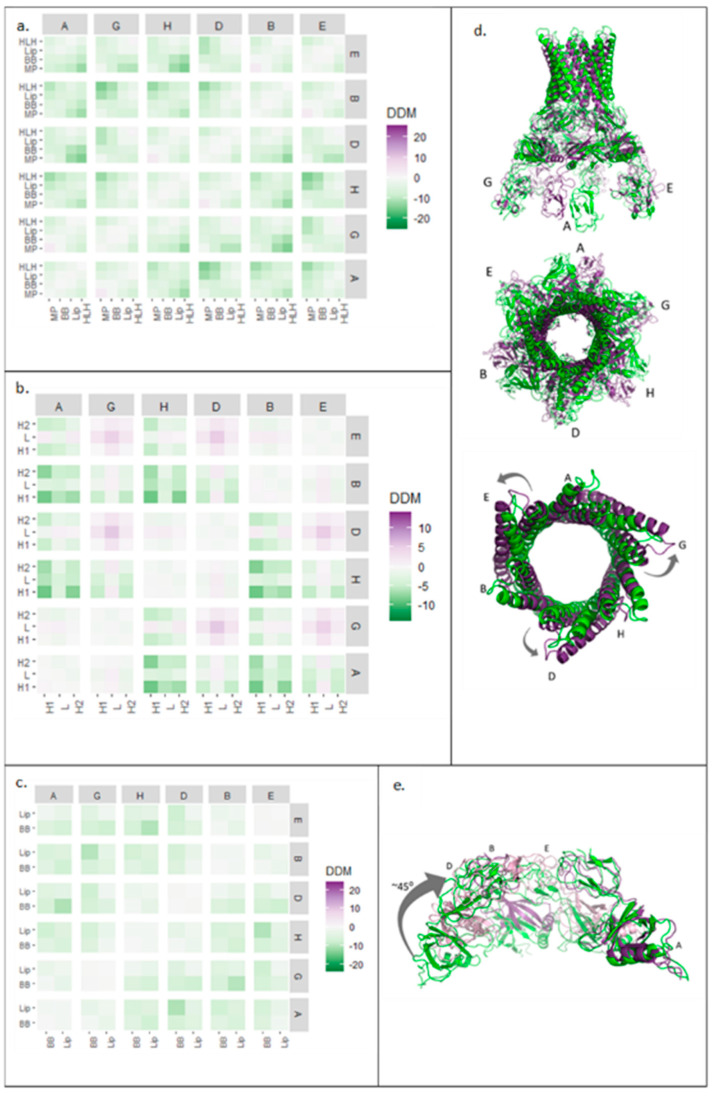
Block difference distance matrix (DDM) analysis of AcrA and HLH, BB, and lipoyl domains in hexameric assembly. (**a**) Average (DDM) changes for all AcrA domains in all six AcrA subunits. (**b**) Average DDM changes for HLH domain in all six AcrA protomers. (**c**) Average DDM changes for lipoyl and BB domains in all six AcrA subunits. (**d**) Resting (green, 5V5S) and transport (purple, 5NG5) structures of AcrA hexamer from the side (top) and above (middle) and of AcrA HLH domains from above (bottom). (**e**) BB and lipoyl domains from four of the six AcrA subunits aligned with subunit A in resting (green, 5V5S) and transport (purple, 5NG5) states. Subunit numbering from PDB: 5NG5. HLH: helix loop helix, BB: beta barrel.

As in the case of TolC, AcrA protomers assemble into a multimeric state in the pump, meriting an analysis of movement in the quaternary structure. A block DDM analysis of the AcrA hexameric ring showed symmetrical changes (Figure 5a,d), including larger changes at the quaternary level than the tertiary. Conformational compaction of the ring (pale green colouring in the distribution) was also observed throughout the plot.

To characterise the changes further, isolated AcrA domains were analysed. The two helices of the HLH domain, within a protomer, showed similar symmetrical movements (Figure 5b,d), indicating that HLH domains moved as a unit. In contrast to the expansion of the HLH domain within a single AcrA subunit (Figure 1b), at a quaternary level, the HLH domains showed a different pattern of movements. HLHs of protomers A, B, and H contracted, whereas HLHs of protomers D, E, and G expanded relative to each other. As illustrated in Figure 5d, the HLH domains moved slightly upon transition from the resting to the transport state, producing an apparent anticlockwise twisting motion.

The lipoyl and BB domains of AcrA were also analysed using the same approach (Figure 5c). The block DDM showed symmetrical, quaternary contraction. The largest contraction was observed in protomers positioned opposite each other in the ring. Superimposed structures (Figure 5e), aligned with one subunit, showed a 45° inward rolling motion.

### 2.5. An Increase in the Interface Surface Area Is Observed in the Transport State

To further characterise conformational changes of AcrA and TolC on pump opening, the interface surface areas of the close (resting) and open (transport) states were analysed as a proxy for binding energy [44]. As the DDM analysis showed that quaternary changes were more pronounced than tertiary changes, differences in interface areas between subunits were measured.

**Figure 6:**
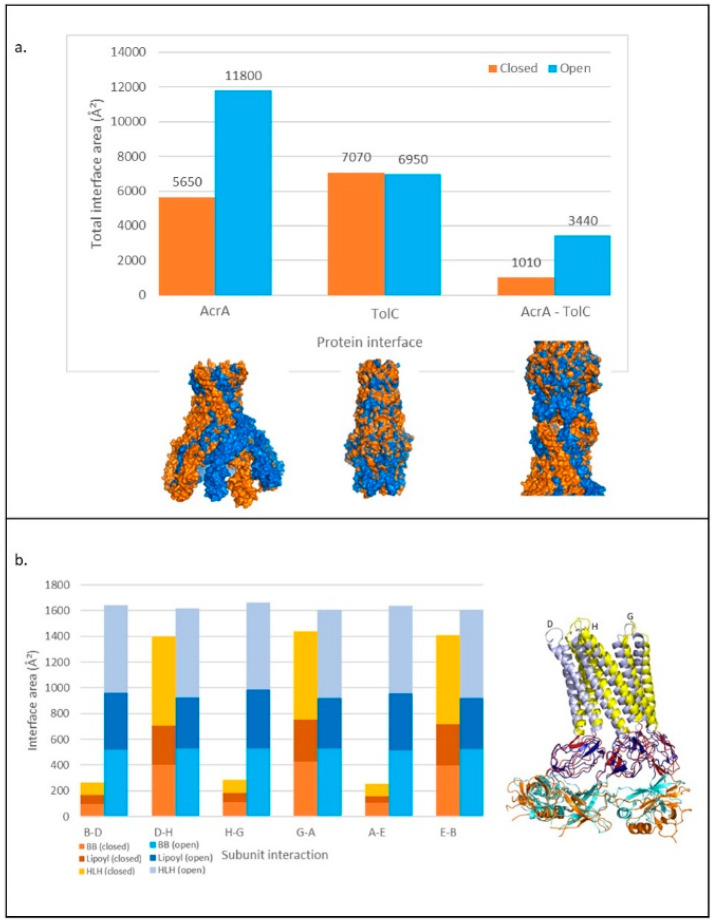
Changes in the interface area within and between TolC and AcrA in transport and resting pump structures. (**a**) Above: Total interface area between subunits and proteins for TolC and AcrA calculated with PDBePISA to three significant figures. Below: Surface of aligned structures of proteins in closed (resting) (orange) and open (transport) (blue) states. (**b**) Left: Change in interface area between AcrA subunits in transport and resting states, showing the contribution of BB, lipoyl, and HLH domains. Right: Three AcrA subunits aligned in resting and transport states. Colours relate to domains on the bar chart. Subunit numbering from PDB: 5NG5. HLH: helix loop helix, BB: beta barrel.

The total interface area between subunits of the AcrA hexamer increased by almost 5000 Å^2^ on transition from the resting to the transport state (Figure 6a). BB, lipoyl, and HLH domain contributions to interface area were calculated separately (Figure 6b). The interface area between domains of adjacent subunits increased on transition from the resting to the transport state for all three domains. We also observed an additional pattern of change: one subunit interface increasing much more than the adjacent interface, i.e., the H-G interface increased dramatically, whilst the D-H interface showed little change (Figure 6b).

For TolC, the total interface between subunits was very similar in both states, with a slightly smaller area in the transport state. As the DDM showed smaller changes for TolC than AcrA (Figure 4a), this was not surprising. However, the tip-to-tip interactions between AcrA and TolC increased by around 2000 Å^2^ on transition from the resting to the transport state. This could be classified as a change from a weak to a strong affinity interaction, with the threshold typically defined as a surface area of more than 2000 Å^2^ [45].

### 2.6. Pattern of Interface Changes Is Conserved in Homologous Pump Assemblies

**Figure 7:**
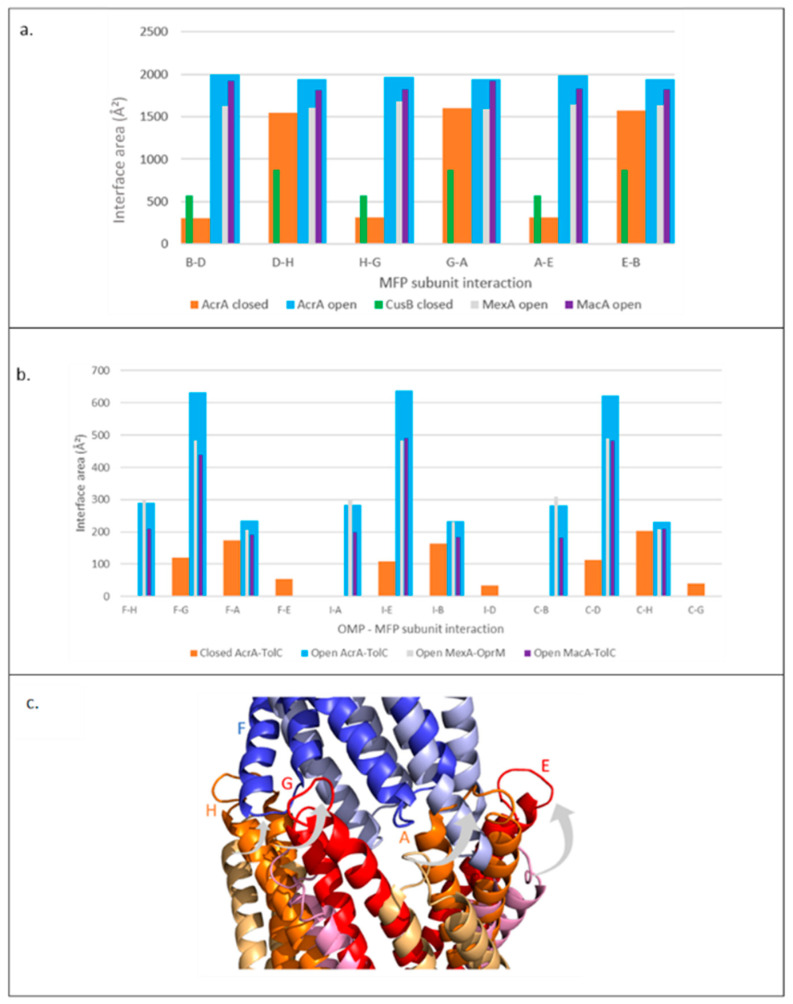
Trends in interface areas in other MFPs. (**a**) Interface area between subunits of different MFPs. (**b**) Tip-to-tip interface areas between OMP periplasmic helices and MFP HLH domains. OMP subunits: F, I, E. MFP subunits: H, G, A, E, B, D. (**c**) Aligned structures of AcrA-TolC tip-to-tip interaction. An individual TolC subunit is shown in the resting (light blue) and the transport (dark blue) state. Four AcrA subunits are shown in the resting (alternate pale yellow and pink) and the transport (alternate orange and red) state. Grey arrows show the direction of AcrA HLH twisting movement on pump activation. Results shown for Open MexAB-OprM (transport state) are from analysis of the 6TA6 PDB file; similar results were obtained for 6IOK and 6IOL as well (data not shown). Subunit numbering is based on AcrA 5NG5. MFP: Membrane Fusion Protein, OMP: Outer membrane protein.

To investigate whether other pump assemblies also exhibit large interface areas in the transport state, we investigated three other MFPs (CusB, MexA, and MacA) (Figure 1c and Figure 7a). CusB structure has only been captured in a resting conformation; in the interface analysis, the interface area between subunits showed a similar pattern to resting AcrA. At all six interfaces, the interface area was relatively small, but the size oscillated between adjacent interfaces. However, this pattern was less pronounced than in resting AcrA and an analysis of a transport structure of CusB would be needed to determine if the interface areas showed a large increase in the transport state. MexA and MacA have only been captured in transport conformations; the interface analysis showed a large interface area that was of similar size to that in the transport state of AcrA. As in AcrA, the interface sizes in MexA and MacA did not oscillate between adjacent interfaces.

We also investigated other pump assemblies to further characterise the MFP-OMP tip-to-tip interactions, assessing the trends and the contributions of individual subunits. The AcrA subunits that contribute to the interaction interface with TolC in the resting state are different to the subunits which interact in the transport state (Figure 7b,c). This is in accordance with the DDM results, where an iris-like opening motion of the HLH domain of AcrA (Figure 5b,d) and the outward movement of the TolC periplasmic helices (Figure 4b) was observed on pump activation. MexA and MacB seemed to form a similar pattern of interactions with their respective OMPs (Figure 7b), suggesting that different MFPs form assemblies through similar interactions.

## 3. Discussion

### 3.1. ECs Maintain Tertiary Fold but Role at Subunit Interfaces Is Less Clear

Previous investigations of sequence covariance in tripartite efflux pumps have provided support for the tip-to-tip interaction between the MFP and the OMP in the pump assembly [34,46], but the relationship between covariance and efflux pump dynamics has not yet been studied. In this work, we contextualised strong ECs with AcrA and TolC protein structures, finding that many strongly coupled residues act to maintain the tertiary fold, as has been extensively reported for many proteins [47,48]. We also identified coupled residues at the interface between TolC subunits (Figure 3a); most of these are physically close, and so are likely involved in maintaining the TolC trimeric assembly. However, one pair, Q164 and A382, was placed around 20 Å apart and so did not form a direct physical interaction, yet was still strongly evolutionarily coupled. This residue pair could potentially be involved in communicating allosteric change. Interestingly, the coupled TolC residue paired in the periplasmic helices, which have been proposed to form an intersubunit hydrogen bonding network to maintain the closed (resting) state of the channel (R367, T152, D153, and Y362 [49]) were not strongly evolutionarily coupled. We can hypothesise that through the transmission of interaction changes between the periplasmic helices upon pump activation, local residue pairs may need to be less strongly evolutionarily coupled, i.e., with various distances distribution thus making them highly dynamic, to maintain interface flexibility and allow communication of conformational changes.

In a similar manner, we found no strong ECs at the interface between AcrA subunits. This may be due to the dynamic nature of the AcrA hexamer, where interface interactions change dramatically in different conformations, thus changing distances between residue pairs [50,51]. In general, we found that whilst direct mapping of ECs onto structures can be useful in the study of protein dynamics, the throughput is too low to investigate large-scale conformational change in macromolecular complexes. One alternative to overcome this could be a vertical approach combining comparison of the modern proteins to their analogues from extinct species of the phylogenetic tree and MD simulations. This could allow to identify crucial residues involved in allosteric effects by narrowing down the number of residues to investigate. Such approach has helped to identify four residues in the β-subunit TrpB of Tryptophan synthase (TS) heterotetrameric αββα complex that are suggested to be essential for the communication between TrpB and the α-subunit TrpA [52].

### 3.2. Movement of TolC Periplasmic Helices and of AcrA BB Domain Appears to Be Evolutionary Conserved

Comparison of the ECs to the DDM analysis for both AcrA and TolC was found to be effective in relating sequence covariance to conformational change. Some areas of movement captured by the DDM, in particular the large outward movement of the HLH of AcrA, did not correlate with the ECs. As discussed above for the AcrA subunit interfaces, the lack of strongly coupled residues may give the domain more flexibility to change conformation, with different residue interactions and various distance distributions forming in the resting and transport states.

However, the DDM analysis also found some regions of movement that did correlate with the ECs (Figure 3b,c). The contraction of the TolC periplasmic helices correlated with some strongly coupled residues. This suggests that the movement, which likely acts to ensure the helices are optimally positioned to interact with AcrA and seal the transport channel, is conserved evolutionarily. The importance of the movement of these helices for channel opening has already been extensively reported [53,54]. In addition, the contraction of the BB domain of AcrA correlated with the ECs data, suggesting this movement is conserved as well. This finding is in accordance with previous work, which has shown that mutations in the AcrA BB domain can affect the BB domain folding in a way that impact AcrA conformational changes that influence interactions with AcrB and TolC and long-range transmission of conformational changes from AcrB to TolC [36,55]. This suggests that this domain could play a central role in long-distance communication. However, closer inspection of the correlation between the ECs and DDM data of the BB domain found the residues that move most are not themselves evolutionarily coupled. This suggests that the more static conserved residues may play a role in promoting the movement of the nearby, more mobile residues which then enable conformational change.

### 3.3. AcrA and TolC Show a Quaternary, Symmetrical Switch on Pump Activation

Through further investigation of the DDM analysis results, we were able to describe efflux pump conformational changes in more detail than by visual comparison of structures alone [13].

In AcrA, the BB domains, which contact AcrB, tightly contract, observed as an inward rolling movement of the BB ring (Figure 5e). This pulls the lipoyl domains to roll and contract, too. As HLH movement follows the BB and lipoyl domains movements, this suggests that the BB and lipoyl movement is communicated to the HLH domains via a flexible linker which pulls the HLH domains towards the lipoyl domains, causing the outward twisting of the HLHs. The HLH domain movement is distinct from the lipoyl domain movement, suggesting that the flexible hinge region between the domains is important, as it can enable such conformational independence. Crystal structures of AcrA indeed suggest a degree of flexibility in this region [50]. Such flexibility is suggested to also be supported by the cellular environment, as Cryo-ET structures of the AcrAB-TolC efflux pump show a rotation of AcrA protomers triggered by the lipid anchorage of AcrA in the inner membrane, leading to TolC opening [16]. In TolC, the DDM analysis showed that the periplasmic helices, in particular H7 (Figure 4b), moved outwards in the transport state while the rest of the TolC channel stayed relatively static, in accordance with previous reports [13]. Mutagenesis studies have shown the importance of the tip-to-tip interactions between the AcrA HLH domains and the TolC periplasmic helices for promoting the widening of the TolC pore [36]. The concerted movements of AcrA and TolC, discernible in detail in DDM results, act to seal the channel to prevent substrate leakage into the periplasm during drug extrusion.

Analysis of AcrA and TolC conformational changes at the tertiary and quaternary levels allowed insight into the key dynamics for pump activation. Although intrasubunit change was observed, for both AcrA and TolC, changes at the quaternary structure level were much more pronounced than at the tertiary level (Figure 4 and Figure 5). Moreover, mutations at the tip of TolC which disrupt efflux pump function can be suppressed by mutations in AcrA, indicating the importance of the AcrA-TolC interface for efflux pump activity [36]. This suggests that changes at subunit interfaces may play an important role in communicating allostery in the pump. Interestingly, the intersubunit changes in AcrA and TolC upon pump activation are symmetrical. As AcrB adopts an asymmetric conformation on substrate binding, communication between AcrA subunits is likely important to ensure that all AcrA protomers, not just those in contact with the substrate-bound AcrB subunit, shift to a symmetrical transport state. The interdomain flexibility of AcrA may be important for this, as MD simulations have previously shown [51], but the DDM analysis in this work suggests that intersubunit flexibility may also play a role. Furthermore, as TolC is also embedded in the peptidoglycan layer, the dynamic of its interaction with the peptidoglycan and surrounding proteins may also affect both the subunits flexibility and the transmission of changes. A lipoprotein, the Braun’s lipoprotein (Lpp), has been found to indirectly interact with AcrAB-TolC via the peptidoglycan layer. Lpp’s role is thought to be important for pump assembly [56].

### 3.4. Increase in Interface Area in Transport State Indicates a Favoured Energy State

Analysis of changes in interface area in AcrA and TolC was performed to investigate energetic transitions between AcrAB-TolC states. The total interface area between AcrA subunits almost doubled from the resting to transport state and the interface area of the tip-to-tip interactions between TolC and AcrA also showed a large increase. The large changes in interface area between subunits were in accordance with the block DDM analysis, which showed contraction at a quaternary level. Taken together, these results indicate that quaternary, rather than intrasubunit, changes may be more significant in the pumping mechanism. Moreover, the increase in interface area suggests that the transport states of AcrA and TolC are at a more favourable energy than their resting states. Although a 10-Å axial contraction on pump activation had already been reported [13], this increase in interface area has not been recognised. The unexpected finding of a predicted lower energy state of AcrA and TolC on pump activation implies that energy must be provided for AcrA and TolC conformational changes to occur when switching back to the resting state.

Expansion of the interface analysis to the structures of homologous efflux pumps found similar patterns of interface changes between the resting and transport states. Of the selected homologues, MexA and AcrA are closely related, but CusB and MacB both belong to different phylogenetic clusters [57], so it is perhaps surprising that all the proteins form very similar interactions within their hexameric rings. However, these findings are in accordance with previous work which has shown the interchangeability of MFPs [14,58]. In addition, analysis of accessible surface area in the *Pseudomonas aeruginosa* pump, MexAB-OprM, found a strong affinity interaction between the MFP, MexA, and the OMP, OprM, in the transport state [28], supporting the findings shown here. The similar pattern of interface changes suggests that the lower energy transport state of the MFP and OMP may be a conserved feature of tripartite efflux pumps.

### 3.5. AcrAB-TolC Conformational Changes Suggest an Allosteric Transport Model

**Figure 8:**
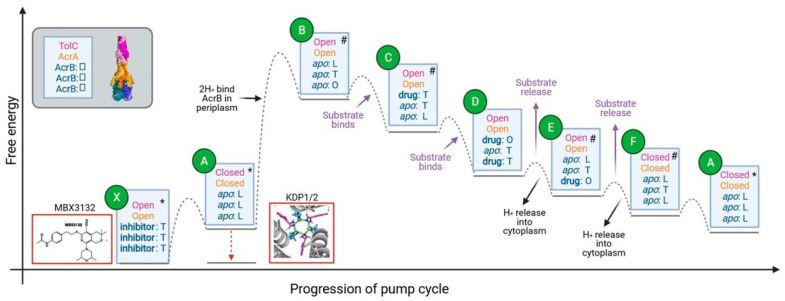
Proposed energy transitions during the AcrAB-TolC pump cycle. Qualitative energy diagram with relative energy levels of predicted states in the pump cycle. Text in blue boxes describes the conformations and the ligand binding states of TolC, AcrA, and the three AcrB subunits from top to bottom. Boxes outlined in red show pump inhibitors. * indicates states where high-resolution structures of the whole pump assembly exist. # indicates where high-resolution structures of AcrB in the specified conformation exist. The dotted lines indicate energy barriers to transition between the connected species, with maxima indicating transition states. AcrB subunit conformations are L = Loose, T = Tight, O = Open.

As discussed above, the open (transport) state of AcrA and TolC appeared to be at a lower energy than the closed (resting) state. This raises the paradox that the pump would be trapped in an energy minimum and not be able to efflux substrate. To reconcile this finding with the logical requirement for energetic transitions during AcrB pump cycling, we propose a model for conformational changes in AcrAB-TolC (Figure 8).

The model shown in Figure 8 shows a simplified description of the energetic transitions during the pump cycle. In the resting assembled pump (state A in Figure 8): the three AcrB subunits are all in the L conformation; AcrA is in an inactive, extended conformation, with an unsealed channel made of asymmetric protomers and the TolC channel pore is closed by the periplasmic helices [13]. Based on our interface area measurements, we propose that the pump subsequently shifts to a more stable transient intermediate state with AcrA and TolC in an open (transport) state and AcrB in an apo LTO state (state B). AcrB’s shift to an LTO conformation on drug binding is thought to be coupled to proton binding to the periplasmic side of AcrB [37]. Therefore, we suggest that proton binding drives the conformational changes of AcrA and TolC to an open conformation. The conformational change of AcrB is communicated to AcrA through the extensive contacts between the docking and pore domains of AcrB and the MP and BB domains of AcrA [13].

On substrate binding to the periplasmic pocket of the AcrB O-state subunit [21], the energy of the pump is likely to be lowered (state C in Figure 8). AcrB will then cycle through conformations to extrude substrates (states C–F), whilst AcrA and TolC are predicted to stay in an open (transport) conformation [13]. Substrates are thought to bind to different AcrB subunits simultaneously and then transition through the same sequence of states: L to T to O, culminating with extrusion. Due to unfavourable energies, two AcrB subunits will never be in the O conformation at the same time [37]. The apparent fixed open conformation of AcrA and TolC during AcrB cycling may be due to the strong tip-to-tip interactions between the proteins seen in the interface analysis and due to the flexibility of AcrA. This flexibility likely acts to buffer the conformational changes of AcrB, preventing these from causing changes to the rest of the pump [50,51]. Structures of almost all of the conformational states of AcrB predicted to occur in the model during pump cycling have already been obtained, giving support to the proposed model. The only state that has not been experimentally observed is OTT. It would be useful to perform MD simulations for the OTT state to predict how energetically favourable this conformation would be.

Release of substrates from AcrB through the AcrA-TolC pore (state E) is predicted to be coupled to cytoplasmic proton release to provide energy for this step [59]. Release of substrates and protons will lower the energy of the pump and so AcrA and TolC are predicted to transition back to the less favourable closed conformation with AcrB in the LLL state (state A).

This allosteric model highlights potential strategies to block pump function by trapping the pump in an energy minimum. The inhibitor MBX3132 is known to bind to the AcrB periplasmic pocket [60] in all three T state subunits with AcrA and TolC in the open (transport) state. The binding of MBX3132 to AcrB is predicted to lower the energy of the inhibitor-bound state (state X) relative to the equivalent apo state (state B). The model also highlights the central role of the PMF in promoting the conformational change needed for pump activation and so targeting the generation or maintenance of the PMF would offer an alternative strategy to inhibit the pump.

Although our diagram in Figure 8 suggests a 1:1 proton: drug stoichiometry, the model does not presuppose a specific ratio. Indeed, some studies suggest that two protons bind per cycle [37], whilst others report that only one is involved [59]. It has been proposed that changes in the pH of the periplasm due to the PMF activity of AcrB could induce AcrA to change conformation directly, and this could act instead of the conformational changes being transduced from AcrB on proton binding [55,61]. However, mutations to AcrB residues involved in the proton-relay network abolish the activity of AcrAB-TolC, despite not causing substantial structural change, indicating the importance of proton binding for the pump’s conformational changes to occur [37,62]. In addition, molecular dynamic simulations of AcrB in the presence of indole (as a substrate of protonated systems) have shown that the protonation of aspartic residues involved in the proton-relay network triggers conformational changes from binding state to extrusion state in one of the three monomers and that all three monomers return to access state in absence of indole [63]. In a similar way, the protonation of proton-relay aspartic pair of MFS transporters is likely to cause conformational changes of the N-terminal transmembrane subdomain towards the cytosolic direction [64]. The pH of the periplasm has been proposed to be buffered [65], which could also impact the extent of AcrA changes. Studies involving AcrB fluorescent substrates have shown that substrate dissociation rates are inversely related to pH, supporting the notion that protonation triggers conformational change [66].

Our model did not consider pump assembly or disassembly. Energy from the PMF is not thought to be required for pump assembly [67], but some in vitro studies suggest that the PMF may promote its disassembly [68,69]. In such case the pump may not cycle repeatedly through the energetic transitions shown in Figure 8, but instead only transition through the states once before disassembling.

### 3.6. The Allosteric Transport Model Might Be Common across Efflux Pumps

The shared pattern of interface changes found in this report, and the previously reported interchangeability of MFPs [14,58], suggest that the manner of energetic transitions proposed here for AcrAB-TolC may be conserved in other tripartite efflux pumps. Necessarily, the details of the conformational changes that occur will vary between pumps. For instance, the MFP of the BesABC RND pump from *Borrelia burgdorferi*, BesA, does not have a HLH domain and is predicted to form weaker interactions with the OMP BesC, with a smaller interaction interface [70]. Therefore, the active transport state of the pump will be less favourable than AcrAB-TolC and the pump may require less energy to move to the active conformation. The RND transporter AdeB from the AdeABC tripartite system of *Acinetobacter baumannii* shows structural features that are distinct from other RND trimeric pumps, as its binding, access, and extrusion states all present with two periplasmic clefts open and one cleft closed [71]. The structure of AdeB in complex with ethidium bromide shows that one protomer can simultaneously accommodate three ethidium bromide molecules and that protomers within AdeB trimer are able to independently export substrates. The substrates also tend to stabilize the trimer assembly, which may facilitate the MFP AdeA binding and substrate extrusion. Structural characterisation of more tripartite pump assemblies in different conformations would provide insight into how the allosteric mechanism of efflux pumps has been conserved and how they have diverged.

Both the ECs and DDM analyses performed here only provide information on pairwise interactions between residues, and as allostery in multi-subunit complexes is likely to be highly complex, approaches such as neural network [72] or elastic network analysis [73] may help to provide further analysis. One other approach could involve the use of a platform for allosteric analysis such as Ohm [74] to predict and map the location of allosteric sites. As with the work presented here, combining different approaches is likely to be the most fruitful way to gain insight into long-distance communication in multi-subunit efflux pumps.

## 4. Materials and Methods

### 4.1. Evolutionary Couplings

Evolutionary couplings data were calculated using the freely accessible Evolutionary Couplings Server (https://v1.evcouplings.org/, accessed on 1 April 2021) [40] queried with TolC and AcrA sequences (UniProt IDs: P02930 and P0AE06, respectively). A percentage of sequence identity is defined to down-weight redundant sequences during couplings calculation, and a value of 0.8 (at least 80% of identity) was used for the clustering threshold. Sequences taken from the UniRef90 database were used for multiple sequence alignments built and the number of sequences to include in the alignment was defined with a bit score range of 0.2 (Higher bitscores (=1) include less sequence in the alignment). Alignment positions and fraction residues (rather than gaps) were included, with a position gap threshold of 0.5 (allowing alignment positions with up to 50% gaps) and a sequence gap threshold of 0.7 (allowing sequences with up to 30% gaps). Pseudo-likelihood maximisation (plmDCA) was used during couplings calculation. Results were plotted using R/ggplot library from the R/tidyverse library used under the R studio environment. Only ECs with cn values more than or equal to 1 were plotted.

### 4.2. Difference Distance Matrix

A custom algorithm for the calculation of DDM was developed in Python programming language; scripts to generate DDM calculations are publicly available on GitHub (https://github.com/malitharatnaonc/differencedistance, accessed on 1 April 2021). Molecular models of the open (transport) and closed (resting) states of the pump (PDB IDs: 5V5S and 5NG5, respectively) were used as input. Outputted data were plotted using the R/reshape2 and R/tidyverse libraries in R studio environment. To perform the block analysis, the mean DDM of the domains were calculated in R studio.

### 4.3. Interface Surface Area

Interface surface areas were calculated using ‘Protein interfaces, surfaces and assemblies’ (PISA) service at the European Bioinformatics Institute, (http://www.ebi.ac.uk/pdbe/prot_int/pistart.html, accessed on 1 April 2021) [41], which measures the difference in total accessible surface areas of isolated and interfacing structures. To measure interface area changes of isolated domains, we generated structure models using Coot or PyMOL that encompass the contacting domains. The following PDB files were used: 5V5S and 5NG5 for the open (transport) and closed (resting) states of AcrAB-TolC, 3NE5 for the closed state of CusAB, 5NIK for the open state of MacAB-TolC, and 6TA6, 6IOK, 6IOL for the open state of MexAB-OprM.

## 5. Conclusions

The allosteric model proposed here for the mechanism of action of tripartite efflux pumps serves as a starting point for future experimental and computational research. Recent work has already found evidence of additional interactions that add complexity to the model for pump allostery. For instance, it has been found that AcrA may act as a necrosignal, binding to the external-facing BB domain of TolC to stimulate pump efflux as an adaptive resistance mechanism [75]. To gain further insight into the allostery of tripartite efflux pumps, a range of biochemical, structural, and bioinformatic approaches will be necessary, and hopefully the complex mechanism of these macromolecular machines will become clearer.

## Data Availability

The data presented in this study are available in the article. Structures used in this study are available in the PDB database (pdb codes: 3NE5, 5V5S, 5NG5, 6TA6, 5NIK, 1A3N and 2DN1), UniRef90 and Uniprot database (accession numbers: P02930 and P0AE06). The freely accessible Evolutionary Couplings Server (https://v1.evcouplings.org/, accessed on 1 April 2021) was used for Evolutionary couplings data calculation.
